# Expression in skin biopsies supports genetic evidence linking *CAMKK2*, *P2X7R* and *P2X4R* with HIV-associated sensory neuropathy

**DOI:** 10.1007/s13365-023-01134-2

**Published:** 2023-05-11

**Authors:** Jessica Gaff, Fitri Octaviana, Connie Jackaman, Peter Kamerman, John Papadimitriou, Silvia Lee, Jenjira Mountford, Patricia Price

**Affiliations:** 1 Curtin Medical School, Curtin University, Bentley, 6102 Australia; 2 Neurology Department, Faculty of Medicine, Universitas Indonesia, Jakarta, Indonesia; 3 Neurology Department, Cipto Mangunkusumo Hospital, Jakarta, Indonesia; 4 Curtin Health Innovation Research Institute, Bentley, Australia; 5 School of Physiology, University of Witwatersrand, Johannesburg, South Africa; 6 Pathwest Laboratories, Perth, Australia; 7 Department of Microbiology, Pathwest Laboratory Medicine, Perth, Australia

**Keywords:** HIV-associated sensory neuropathy, CaMKK2, P2X7R, P2X4R, Intraepidermal nerve fibre density

## Abstract

HIV-associated sensory neuropathy (HIV-SN) affects 14–38% of HIV+ individuals stable on therapy with no neurotoxic drugs. Polymorphisms in *CAMKK2*, *P2X7R* and *P2X4R* associated with altered risk of HIV-SN in Indonesian and South African patients. The role of CaMKK2 in neuronal repair makes this an attractive candidate, but a direct role for any protein is predicated on expression in affected tissues. Here, we describe expression of CaMKK2, P2X7R and P2X4R proteins in skin biopsies from the lower legs of HIV+ Indonesians with and without HIV-SN, and healthy controls (HC). HIV-SN was diagnosed using the Brief Peripheral Neuropathy Screen. Biopsies were stained to detect protein gene product 9.5 on nerve fibres and CaMKK2, P2X7R or P2X4R, and were examined using 3-colour sequential scanning confocal microscopy. Intraepidermal nerve fibre densities (IENFD) were lower in HIV+ donors than HC and correlated directly with nadir CD4 T-cell counts (*r* = 0.69, *p* = 0.004). However, IENFD counts were similar in HIV-SN+ and HIV-SN− donors (*p* = 0.19) and so did not define neuropathy. CaMKK2+ cells were located close to dermal and epidermal nerve fibres and were rare in HC and HIV-SN− donors, consistent with a role for the protein in nerve damage and/or repair. P2X7R was expressed by cells in blood vessels of HIV-SN− donors, but rarely in HC or HIV-SN+ donors. P2X4R expression by cells in the epidermal basal layer appeared greatest in HIV-SN+ donors. Overall, the differential expression of CaMKK2, P2X7R and P2X4R supports the genetic evidence of a role for these proteins in HIV-SN.

**Supplementary Information:**

The online version contains supplementary material available at 10.1007/s13365-023-01134-2.

## Introduction

HIV-associated sensory neuropathy (HIV-SN) is a debilitating complication of HIV infection and antiretroviral therapy (ART) (Affandi et al. 2008; Wadley et al. 2011; Octaviana et al. 2018; Pillay et al. 2019). Despite the use of ART that excludes drugs associated with neurotoxicity (usually stavudine) in the last decade, HIV-SN continues to impact up to 38% of people living with HIV (PLWH) (Octaviana et al. 2018; Pillay et al. 2019). Symptoms of HIV-SN include numbness, “pins and needles”, disordered sensation and neuropathic pain, which impact an individual’s ability to work and their quality of life (Phillips et al. 2010). No interventions prevent or reverse HIV-SN progression (Phillips et al. 2010), so a better understanding of underlying mechanisms is sought.

Clinical features of HIV-SN include degeneration of long axons in a “die-back” manner, so that reduced intraepidermal nerve fibre densities (IENFD) have been observed in some studies describing PLWH with severe HIV disease and/or experiencing pain (Polydefkis et al. 2002; Phillips et al. 2014). Increased infiltration of mononuclear cells and cytokine expression in dorsal root sensory ganglia (DRG) (Jones et al. 2005; Hahn et al. 2008) and increased expression of chemokine receptors by inflammatory cells surrounding intraepidermal nerves (Mountford et al. 2018) suggest a central role for inflammation in the manifestation of HIV-SN. An inflammatory aetiology is supported by genetic associations with polymorphisms in a block of genes surrounding the *TNF* gene (Wadley et al. 2015; Gaff et al. 2020b), and in three neighbouring genes: *P2X7R*, *P2X4R* and *CAMKK2* (the *P2X*-block) (Goullee et al. 2016; Gaff et al. 2019, 2020a; Safri et al. 2020). We linked polymorphisms and haplotypes from the P2X-block with HIV-SN in Indonesian and South African patients, implicating the encoded proteins in the pathogenesis of HIV-SN (Gaff et al. 2019, 2020a). Whilst the clearest associations were with CAMKK2, linkage disequilibrium makes a role for P2X4R or P2X7R plausible. These proteins are the focus of the present study.

*CAMKK2* encodes calcium/calmodulin-dependant protein kinase kinase 2 (CaMKK2) which phosphorylates adenosine monophosphate (AMP)-activated protein kinase (AMPK), sirtuin 1 (SIRT1), and calcium/calmodulin-dependant kinase 1 and 4 (CaMKIV and CaMKI) (Kokubo et al. 2009; Racioppi and Means 2012; Wen et al. 2013). AMPK activation mediates inflammation and apoptosis (Racioppi et al. 2012; Zhang et al. 2019). SIRT1 activation regulates axonal regeneration, promotes dendrite arborisation and protects neurons from oxidative stress (Li et al. 2008; Codocedo et al. 2012; Liu et al. 2013). CaMKIV activation upregulates nuclear factor kappa B and cyclic AMP response element-binding protein which stimulates brain-derived neurotrophic factor (BDNF), promoting neuronal growth and survival (Cao and DeLeo 2008; Wayman et al. 2008; Racioppi and Means 2012). Excessive BDNF expression is implicated in neuropathic pain (Ulmann et al. 2008). Activated CaMKI plays a role in axonal growth cone morphology and outgrowth, dendrite arborisation and synapse formation (Wayman et al. 2004; Ageta-Ishihara et al. 2009).

*P2X7R* and *P2X4R* encode purinergic P2X receptors 7 and 4 (P2X7R and P2X4R, respectively) which are activated by adenosine triphosphate (ATP) and are involved in inflammatory and neurotransmission pathways (Makoto et al. 2012). Activation of P2X7R in microglia in spinal dorsal horn and satellite glial cells in DRG induces pro-inflammatory interleukin-1 beta (IL-1β), IL-6 and tumour necrosis factor-alpha (TNFα) via p38-mitogen activated protein kinase (p38-MAPK) (Guo et al. 2020). Mice treated with P2X7R antagonists display reduced expression of IL-1β and IL-6 and alleviated mechanical allodynia in a neuropathic pain model (Chessell et al. 2005). P2X4R is also implicated in the development of neuropathic symptoms in rodent models (Tsuda et al. 2003). Intraspinal administration of P2X4R antisense oligodeoxynucleotides reduced P2X4R expression and inhibited tactile allodynia following nerve injury, and intraspinal administration of P2X4R-positive microglia triggered tactile allodynia (Tsuda et al. 2003). Upregulation of P2X4R results in p38-MAPK-dependent release of BDNF, IL-1β, TNFα and IL-6, leading to neuropathic pain (Ulmann et al. 2008; Zhang et al. 2020).

Experimental evidence implicates P2X7R, P2X4R and CaMKK2 expressed by cells in DRG, spine and brain in neurological outcomes affecting the periphery. It is also plausible that these proteins may contribute directly to the pathogenesis of HIV-SN via degeneration of sensory nerve terminals in the skin. P2X7R is expressed in the skin by keratinocytes, Langerhans cells, dermal dendritic cells, T-cells and macrophages (Geraghty et al. 2016). P2X4R expression has been detected in cultured keratinocytes, macrophages and sensory axon terminals (Inoue et al. 2007; Gaff et al. 2018; Moehring et al. 2018; Sadler et al. 2020), and CaMKK2 is expressed by monocytes/macrophages (Racioppi and Means 2012; Gaff et al. 2018).

Here, we assess ex vivo expressions of CaMKK2, P2X7R and P2X4R and their association with nerve fibres in skin biopsies from the lower leg, donated by Indonesian PLWH with and without HIV-SN, and healthy controls (HC).

## Materials and methods

### Participants and phenotyping

The study was approved by the Ethics Committee of the Faculty of Medicine, Universitas Indonesia (579/UN2.F1/ETIK/2014), and validated by Curtin University (HR210-2015). Written and informed consent was obtained from all participants. HIV+ adults who had used ART for at least 12 months (median = 6.4 years; range = 1.2–11.7 years), but who had never been exposed to stavudine, were screened for neuropathy at POKDISUS HIV Care Clinic, Cipto Mangunkusumo Hospital, Jakarta, Indonesia, in 2016 (Octaviana et al. 2018). Individuals with a history of other conditions potentially associated with neuropathy or conditions preventing informed consent were excluded. Neuropathy was assessed using the Brief Peripheral Neuropathy Screen (BPNS). HIV-SN was defined as present with one or more lower limb neuropathic symptoms (pain, aching or burning, “pins and needles” or numbness) and reduced ankle reflexes or vibration sense at the great toe (128-Hz tuning fork vibration felt for 10 s or less). Neuropathy was not diagnosed in patients with only asymptomatic neuropathic signs, as the presence of both symptoms and signs better associates with impaired peripheral nerve function and pathology (Cherry et al. 2005; Lauria et al. 2010). Biopsies were collected from six individuals with HIV-SN (HIV-SN+) and six individuals without HIV-SN (HIV-SN−) to provide sufficient tissue to assess expression of P2X4R, P2X7R and CaMKK2 in triplicate from three individuals per group. Five control biopsies were provided by healthy, age-matched adults of South East Asian ancestry (HC) who declared no risk factors for HIV.

### Biopsy collection

Biopsies were collected from the lower leg as published previously (Mountford et al. 2018). Briefly, 2% lidocaine with epinephrine anaesthesia was injected subcutaneously approximately 10 cm above the lateral malleolus. Biopsies were collected using a 3-mm circular skin punch, fixed in 4% paraformaldehyde-lysine-periodate overnight at 4 °C, and incubated with cryoprotectant (20% glycerol, 80% 0.1 M Sorenson’s phosphate buffer) overnight at 4 °C prior to storage at −20 °C. Biopsies were sectioned at 50 μm using a freeze cryostat sliding microtome (Leica Biosystems, Nussloch, Germany) and stored in antifreeze (33% glycerol, 33% ethylene glycol, 10% 2 × phosphate buffer, 24% dH_2_O) at −20 °C.

### Immunofluorescent protocol

Floating sections were incubated with Image-iT FX Signal Enhancer (Invitrogen, Carlsbad, CA, USA) for 30 min at room temperature, incubated over consecutive nights at 4 °C with primary, secondary, and detection antibodies diluted in Tris-buffered saline (TBS) with 5% normal donkey serum (NDS), and washed the following morning six times for 1 h at room temperature using TBS with 0.01% v/v triton-X (TBS-wash). Sections were treated with goat anti-CaMKK2 (sc-9629; 2 μg/ml; Santa Cruz Biotechnology, Dallas, TX, USA), anti-P2X4R (ab134559; 5 μg/ml; Abcam, Cambridge, UK) or anti-P2X7R (ab105047; 5 μg/ml; Abcam), followed by biotinylated donkey anti-goat IgG (ab6884; 20 μg/ml; Abcam) and detected with streptavidin conjugated to AlexaFluor™ 647 (S-32357; 20 μg/ml; Invitrogen). Protein gene product 9.5 (PGP9.5) was targeted using rabbit anti-PGP9.5 (ab15503; 2 μg/ml; Abcam) and detected with mouse anti-rabbit IgG conjugated with Dylight^®^ 594 (ab96893; 5 μg/ml; Abcam). Nuclei were stained with 4′,6-diamidino-2-phenylindole (DAPI; 1:10,000; Invitrogen) for 10 min at room temperature to visualise tissue morphology. Sections were washed twice with TBS-wash, and mounted on glass slides (Proscitech, Queensland, Australia) with a coverglass (#1.5; Proscitech) using Immumount (Thermo Scientific, Waltham, MA, USA). Sections treated without primary antibodies were included as negative controls (Supplementary Fig. 1).

### Microscopy

Samples were imaged using an inverted Nikon A1+ confocal microscope (Nikon Instruments, New York, NY, USA). Images were acquired using a digital scan resolution of 0.64 μM/pixel, pinhole of 1.2 airy units and pixel dwell 6.2 with 1024 resolution using a 20 × Plan Apo dry objective (N.A. 0.75; Olympus Corporation, Tokyo, Japan). Sequential scanning was completed using three lasers: 405 nm (450/50 filter), 561 nm (595/50 filter) and 640 nm (700/75 filter) to view nuclei, PGP9.5 + nerve fibres and P2X4R+ , P2X7R+ and CaMKK2+ cells, respectively. At least three images spanning 0.5 mm were acquired from every section across the full width of the epidermis in a z-series at 1.1-μm intervals (Nyquist settings) through the full thickness of the section. Z-series were combined for each section into a maximum intensity projection image with NIS-Elements Viewer (Nikon Instruments). Equivalent thresholds were applied across all images to assess PGP9.5+ nerve fibres (red) and P2X7R-, P2X4R- or CaMKK2-positive cells (green).

### CaMKK2, P2X7R and P2X4R expression and intraepidermal nerve fibre density

Expression patterns of CaMKK2, P2X7R or P2X4R and association with PGP9.5+ nerve fibres were investigated in a subset of HC (*n* = 3), HIV-SN− (*n* = 3) and HIV-SN+ (*n* = 3) donors selected randomly for each protein. A minimum of three images per section from three sections per donor were collected and expression was described and semi-qualitatively assessed by a single rater (JG) blinded to participant diagnoses. All CaMKK2+ and P2X7R+ cells were counted. Most cells in the basal layer of the epidermis were P2X4R+ and therefore were scored using a brightness scale: 1, very weak; 2, weak; 3, moderate; 4, strong; 5, very strong (Supplementary Table 1).

The IENFD was determined from three images from all HC (*n* = 5), HIV-SN− (*n* = 6) and HIV-SN+ (*n* = 6) donors. Images were available for an additional two HC, three HIV-SN− and two HIV-SN+ donors assessed for IENFD in 2017 using the same study criteria and protocols, and were included here (Mountford et al. 2018; Octaviana et al. 2018). All images were de-identified and PGP9.5+ nerve fibres were quantitated by three raters (JG, PK and PP) blinded to donor diagnoses. Quantitation was completed using standard IENFD protocols (Lauria et al. 2010) in which single fibres crossing the dermo-epidermal junction are counted and secondary branches excluded. Average counts were doubled to generate IENFD per square millimetre. Fisher’s exact tests, Mann–Whitney tests and Spearman’s correlations were used as appropriate to compare IENFD with donor characteristics using GraphPad Prism version 8.2.1 for Windows (Graphpad Software, La Jolla, CA, USA). Intraclass correlation coefficients were calculated (two-way random, single measures and absolute agreement) using the “irr” package (Gamer et al. 2010) in the R statistical environment (R Core Team 2010).

## Results

### Intraepidermal nerve fibre densities correlated with nadir CD4 T-cells/μl but did not differ between donors with and without HIV-SN

Confocal images of PGP9.5+ intraepidermal nerve fibres were collected (Supplementary Fig. 2) and IENFD determined for all donors (Table 1). The intraclass correlation coefficient was 0.86 (95% confidence interval = 0.79–0.92), suggesting strong agreement between raters (data not shown). IENFD was significantly higher for HC than for those with HIV-SN (*p* = 0.02) and higher than for those without HIV-SN but this difference was not statistically significant (*p* = 0.07). IENFD was similar in HIV-SN+ and HIV-SN− donors (*p* = 0.19; Table 1). There were no differences in age, height, time on ART and current CD4 T-cells between HIV-SN− and HIV-SN+ donors (Table 1; Supplementary Table 2). However, nadir CD4 T-cell counts were lower in HIV-SN+ individuals than in those without HIV-SN (*p* = 0.05) and IENFD correlated positively with nadir CD4 T-cell counts (*r* = 0.67, *p* = 0.004; Supplementary Fig. 3). Age did not correlate significantly with IENFD (*r* =  −0.36, *p* = 0.08). Thus, IEFND associates with the severity of HIV disease and is not a useful marker of neuropathy in our cohort.

**Table 1:** Summary of donor characteristics

**BPNS diagnosis**	**Male**	**Age (years)**	**Height (cm)**	**Time on ART (months)**	**Nadir CD4 (cells/μl)**	**Current CD4 (cells/μl)**	**IENFD (per mm**^**2**^)
HC	2/7	33 (23–41)	-	-	-	-	12.7 (7.4–17.3)
HIV-SN−	4/9	36 (25–44)	165 (150–179)	58.6 (14.8–141)	225 (6–330)	435 (84–693)	5.2 (3.5–18.4)
HIV-SN+	4/8	36 (29–47)	167 (155–175)	84.4 (24.4–131)	47 (17–166)	485 (284–729)	3.8 (1.3–15.4)
	*p* = 0.99^a^	*p* = 0.83^b^	*p* = 0.63^b^	*p* = 0.33^b^	***p* = 0.05**^b^	*p* = 0.67^b^	*p* = 0.19^b^

Results are presented as median (range)

*BPNS* brief peripheral neuropathy screening tool, *ART* antiretroviral therapy, *IENFD* intraepidermal nerve fibre density

^a^Fisher’s exact test (HIV-SN+ versus HIV-SN−)

^b^Mann-Whitney test (HIV-SN+ versus HIV-SN−)

### Donors with HIV-SN had more CaMKK2+ cells than HIV-SN− donors, and CaMKK2+ cells were close to dermal and epidermal nerves

**Fig. 1:**
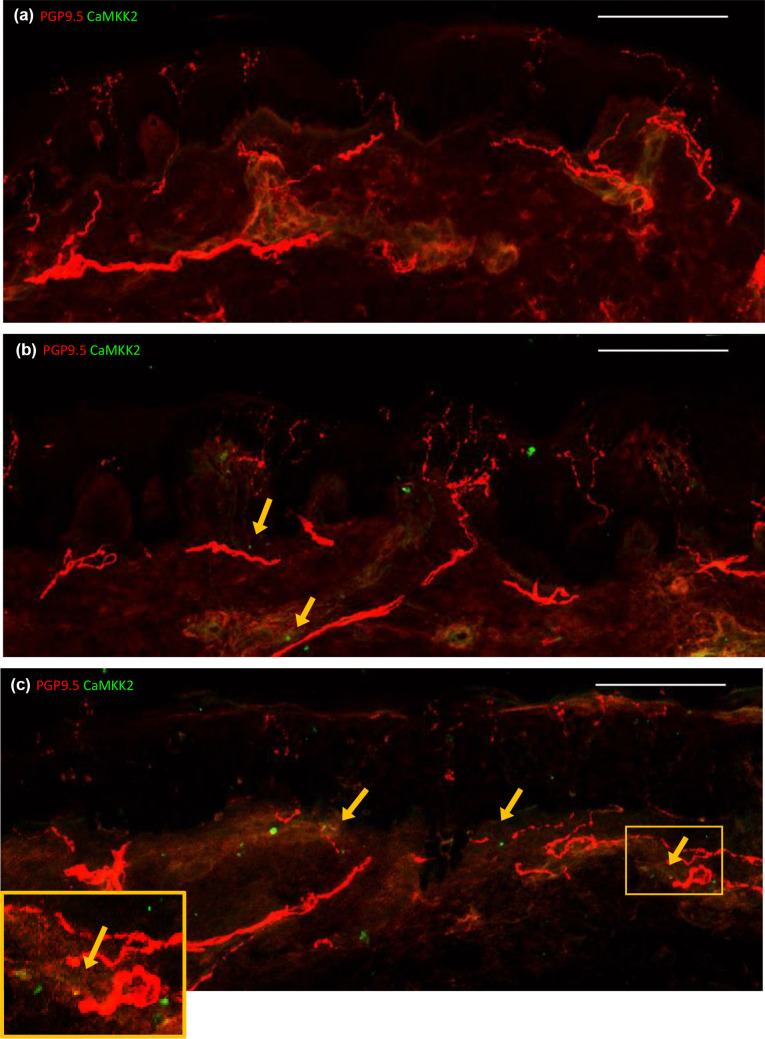
Representative confocal images showing intraepidermal expression of PGP9.5 (red) and CaMKK2 (green) in HC (**a**), HIV-SN– (**b**), HIV-SN+ (**c**). CaMKK2+ cells are very rarely seen in sections from HC donors (**a)** but are observed in sections from HIV-SN– and HIV-SN+ donors (**b**-**c**). CaMKK2+ cells are usually located close to nerves (yellow arrows; b-c) or colocated with nerves - appearing yellow (yellow box; c). scale bar = 100 µm

CaMKK2+ cells were only observed in sections from two of the three HC donors, with expression limited to a few cells (medians of 2 and 2 positive cells per donor; Fig. 1a; Supplementary Table 1). CaMKK2+ cells were common in sections from all HIV-SN− and HIV-SN+ donors. However, HIV-SN− donors had slightly fewer CaMKK2+ cells (medians of 3, 5 and 8 positive cells per donor; Fig. 1b) than HIV-SN+ donors (medians of 8, 14 and 19 positive cells per donor; Fig. 1c). CaMKK2+ cells were typically located close to dermal and epidermal nerves (yellow arrows) or co-located with dermal nerves (yellow box; Fig. 1c), so CaMKK2+ cells may interact with peripheral nerves in HIV+ patients.

### P2X7R+ cells were observed in dermal blood vessels of HIV-SN− donors, but rarely in HC or HIV-SN+ donors

**Fig. 2:**
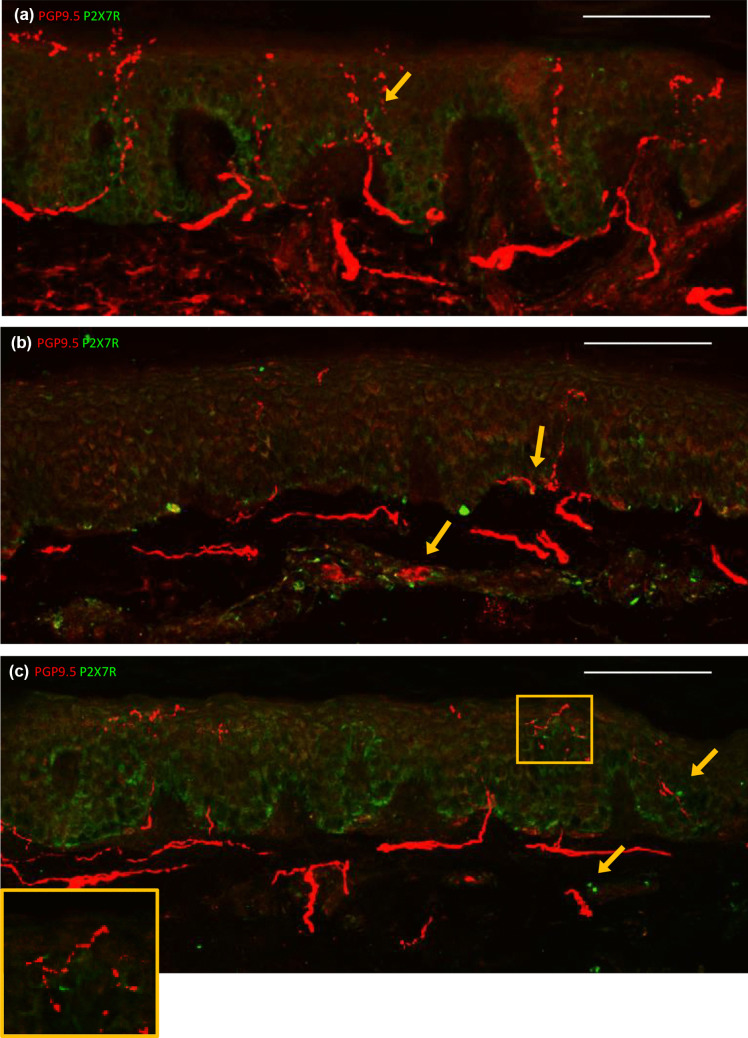
Representative confocal images showing intraepidermal expression of PGP9.5 (red) and P2X7R (green) in HC (**a**), HIV-SN– (**b**), HIV-SN+ (**c**). P2X7R+ cells (yellow arrows) are rarely seen in sections from HC donors (**a**). P2X7R+ cells (yellow arrows) are abundant in dermal blood vessels and sometimes closely located to epidermal nerves of sections from HIV-SN– donors (**b**). P2X7R+ cells are occasionally seen in dermal blood vessels and closely located to epidermal nerves (yellow box) in HIV-SN+ donors (**c**). scale bar = 100 μm

P2X7R was variably expressed across donor groups but was rare in sections from HC (medians of 1, 2 and 4 positive cells per donor; Fig. 2a; Supplementary Table 1). Expression was also rare in sections from HIV-SN+ donors, but a few P2X7R+ cells were observed in dermal blood vessels (yellow arrows) and some within the epidermis close to epidermal nerves (yellow box; medians of 4, 4 and 6 positive cells per donor; Fig. 2c). Sections from two of the three HIV-SN− donors exhibited an abundance of P2X7R+ cells in dermal blood vessels (medians of 18 and 20 positive cells per donor; Fig. 2b). Sections from the remaining HIV-SN− donor had moderately higher levels of P2X7R expression in blood vessels than HC and HIV-SN+ donors (Supplementary Tables 1 and 2, donor #11). P2X7R+ cells were observed close to epidermal nerve fibres in sections from all three HIV-SN− donors (Fig. 2b). This may suggest upregulation by HIV disease, potentially as a mechanism that suppresses or prevents HIV-SN.

### P2X4R expression may be increased in the epidermis of HIV-SN+ donors

**Fig. 3:**
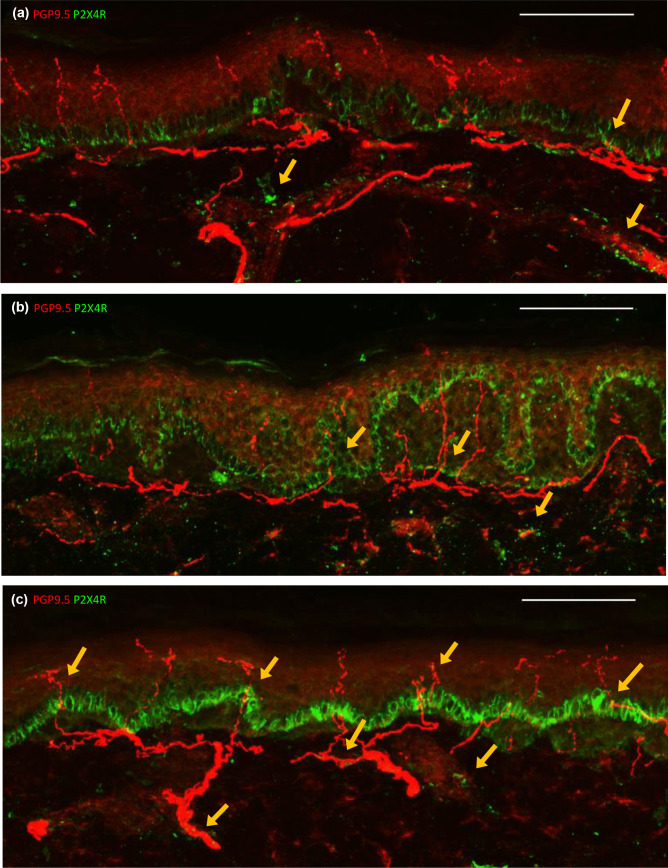
Representative confocal images demonstrating intraepidermal expression of PGP9.5 (red) and P2X4R (green) in HC (**a**), HIV-SN– (**b**), HIV-SN+ (**c**). P2X4R expression is observed in the basal layer of the epidermis and dermal blood vessels of all donors but was increased in sections from donors with HIV-SN (**c**). P2X4R+ cells were observed in dermal blood vessels in all donors and located near dermal and epidermal nerves (yellow arrows). scale bar = 100 μm

P2X4R+ cells were present in dermal blood vessels and basal layer of the epidermis in all sections from all three HC, HIV-SN− and HIV-SN+ donors (Fig. 3; Supplementary Table 1). P2X4R+ cells were often closely located to epidermal nerves and sub-epidermal nerve plexi (yellow arrows; Fig. 3a–c). Whilst P2X4R+ cells occurred in all donors, its expression in the basal layer of the epidermis was more abundant in sections from all three HIV-SN+ donors (median brightness scores of 4, 4 and 5; Fig. 3c). compared to HC (median brightness scores of 1, 2 and 3; Fig. 3a) and HIV-SN− donors (median brightness scores of 2, 2 and 3; Fig. 3b). The trend is consistent with a role for P2X4R in HIV-SN.

## Discussion

We assessed PGP9.5+ IENFD and expression of P2X7R, P2X4R and CaMKK2 in skin biopsies from healthy controls, and HIV-SN− and HIV-SN+ Indonesians receiving stavudine-free ART for > 12 months. The median (range) IENFD for HC was 12.7 (7.4–17.3/mm^2^), consistent with healthy individuals in published studies of other peripheral neuropathies (McArthur et al. 1998; Bakkers et al. 2009). IENFD was lower in HIV+ donors but similar in donors with and without HIV-SN (Table 1). We are not the first to show this. A longitudinal investigation of 150 HIV+ Thai individuals found no difference in IENFD between those with and without HIV-SN irrespective of the use of stavudine (Shikuma et al. 2015). Lower IENFD was recently associated with the presence of neuropathic pain at the biopsy site (Patel and Kamerman 2019). The prevalence of neuropathic pain in Indonesian HIV-SN+ patients is low (5/28; (Octaviana et al. 2019)) and pain was not reported by patients described here.

Age is negatively correlated with IENFD in healthy individuals (Bakkers et al. 2009) but has not been linked in HIV patients (Polydefkis et al. 2002; Phillips et al. 2014; Shikuma et al. 2015; Mountford et al. 2018). Here, HIV+ participants are relatively young (aged 23 to 47) and the age-associated decline in IENFD was not statistically significant (*r* =  −0.36, *p* = 0.08). However, we identified a positive correlation between nadir CD4 T-cell counts and IENFD in the HIV+ groups (*r* = 0.67, *p* = 0.004; Supplementary Fig. 3). We previously associated lower CD4 T-cell counts at the time of assessment with greater risk of HIV-SN in Indonesians (Octaviana et al. 2018), and with lower nadir CD4 T-cells in HIV+ Africans (Gaff et al. 2020a). A lower CD4 T-cell count reflects greater severity of HIV disease, supporting a direct role for HIV itself in the degeneration of epidermal nerve fibres. Early initiation of ART is essential to minimise HIV disease, and may preserve IENFD. CaMKK2+ cells were identified in all HIV+ sections. CaMKK2+ cells were usually located close to or co-located with damaged nerve fibres and were more common in donors with HIV-SN (Fig. 1). CaMKK2 activates AMPK, a master regulator of cellular energy homeostasis. AMPK activation can replenish ATP supplies required for energy-intensive axonal growth by recruiting mitochondria to the site of repair (Sheng 2017). Therefore, close association between CaMKK2+ cells and nerve fibres may reflect CaMKK2-mediated neuronal growth and repair pathways. CaMKK2 is also expressed by macrophages and mediates inflammatory pathways (Racioppi and Means 2012; Gaff et al. 2018). CaMKK2 activates CaMKIV which in turn activates the p38-MAPK cascade and activation factor 1 (AP-1), inducing pro-inflammatory cytokines including TNFα, IL-1β and IL-6 (Ageta-Ishihara et al. 2009). It is plausible that CaMKK2+ cells may contribute directly to a reduced IENFD and HIV-SN via neuronal or inflammatory pathways.

P2X4R expression was observed in the basal layer of the epidermis, in blood vessels and closely located to epidermal nerves and sub-epidermal nerve plexi in all donors assessed (Fig. 3). However, P2X4R appeared upregulated in the basal layer of the epidermis in HIV-SN+ donors. Most cells in the basal layer proliferate and differentiate into keratinocytes which can express sensory receptors and produce neuroactive molecules that can illicit nociceptive responses in epidermal axon terminals of sensory neurons in response to noxious stimuli (Talagas et al. 2020). Rodent studies show that mechanical, cold and heat stimulation of keratinocytes produces ATP which activates P2X4R receptors on sensory neurons and results in behaviours associated with temperature stress and pain (Moehring et al. 2018; Sadler et al. 2020). As extracellular ATP can act on P2X4R in an “autocrine” fashion (Di Virgilio and Sarti 2018), the increased expression of P2X4R in the epidermis of HIV-SN+ donors could plausibly exacerbate this nociceptive pathway and contribute to neuropathic symptoms.

P2X4R is also a moderator of inflammation (Ulmann et al. 2008). Sciatic nerve injury in a rodent model of neuropathy upregulates P2X4R expression in spinal microglia leading to increased production of IL-1β, TNFα and IL-6 (Ulmann et al. 2008; Zhang et al. 2020). Activation of keratinocyte P2X4R is associated with production of IL-6 (Inoue et al. 2007). Like P2X4R, P2X7R is implicated in inflammation associated with neuropathic pain (Chessell et al. 2005). Interestingly, P2X7R was abundantly expressed in the blood vessels of HIV-SN− donors, but less in HC or HIV-SN+ individuals (Fig. 2). Expression of P2X7R may depend on the disease or stage, and tissue or cell type (Liu et al. 2016; Amadio et al. 2017). For example, P2X7R expression was upregulated in peripheral blood monocytes and lymphocytes from patients with neuropathic pain, but not patients with chronic nociceptive lower back pain (Luchting et al. 2016). Furthermore, P2X4R and P2X7R expression may be compensatory. P2X4R was upregulated in CD4 T-cells from P2X7R knockout mice in a model of heart transplantation (Vergani et al. 2013).

Our study has limitations. Firstly, we acknowledge the modest number of donors. However, a well-established protocol for determining IENFD (Cherry et al. 2005; Lauria et al. 2010) and assessment of IENFD by multiple raters blinded to donor diagnoses ensured reliable quantification. This was supported by an intraclass correlation coefficient of 0.86 (95% confidence interval = 0.79–0.92) indicating strong agreement between raters. Furthermore, expression of CaMKK2, P2X7R and P2X4R was sought in at least three sections per donor and in at least three images per section so at least nine images per donor were assessed. Further investigations in larger, longitudinal cohorts are required to confirm the expression patterns of CaMKK2, P2X7R and P2X4R but the findings described here were consistent between donors (Supplementary Table 1). Secondly, we utilised a simple clinical tool to diagnose HIV-SN. It is possible that HIV-SN− donors may have had sub-clinical peripheral nerve pathology or physiological malfunctions not detected by this tool. We have also investigated large and small fibre neuropathy diagnosed using established nerve conduction and stimulated skin wrinkling tests, respectively (Safri et al. 2020). IENFD did not differ between donors with and without large or small fibre neuropathies (0.28 and *p* = 0.62, respectively; Supplementary Table 3). No data pertaining to the duration of neuropathy were available due to the cross-sectional design.

In conclusion, we have extended our genetic studies linking alleles of *CAMKK2*, *P2X7R* and *P2X4R* with HIV-SN, providing evidence of differential expression of the encoded proteins in affected tissues. Using multiple sections from three individuals per category, we showed that expression of P2X4R in epidermal basal layer cells was greater in HIV+ donors with HIV-SN, whereas P2X7R+ cells were more abundant in the blood vessels of HIV+ donors without HIV-SN. Moreover, tissue from donors with HIV-SN contained a few more CaMKK2+ cells than donors without HIV-SN, and most CaMKK2+ cells were located near or co-located with PGP9.5+ nerves. This would be consistent with a role for CaMKK2 in the repair of nerves damaged by HIV. Hence, the expression and location of CaMKK2+ , P2X7R+ and P2X4R+ cells support roles for these proteins in the pathogenesis of HIV-SN in patients treated with stavudine-free ART. Our data suggests that CaMKK2 should be prioritised in studies of larger cohorts with follow-up to assess changes over time. Ideally, patients willing to undergo biopsies should also be selected by carriage of polymorphisms or haplotypes associated with risk or protection. The identification of proteins contributing to the development of HIV-SN is an important step in the development of targeted diagnostic and therapeutic strategies. We have provided some interesting candidates.

## Supplementary Information

Below is the link to the electronic supplementary material.

Supplementary file1 (DOCX 2290 KB)

## Data Availability

Data are available from the authors upon request.
