# Supplementary material for: Expression in skin biopsies supports genetic evidence linking CAMKK2, P2X7R and P2X4R with HIV-associated sensory neuropathy
Source: J Neurovirol. 2023 May 11;29(3):241–51. doi: 10.1007/s13365-023-01134-2 (PMC10404215; doi:10.1007/s13365-023-01134-2)
Supplement: Supplementary file 1 — Supplementary file1 (DOCX 2290 KB) [file 13365_2023_1134_MOESM1_ESM.docx]

**Online Supplements**

| 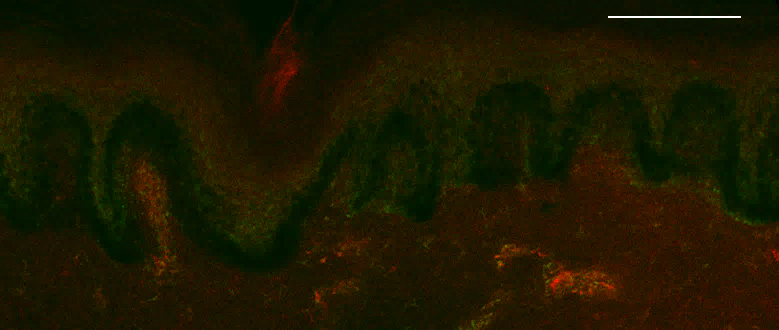  (b) P2X4R **DyLight® 594 AlexaFluor^TM^ 647**  (a) P2X7R **DyLight® 594 AlexaFluor^TM^ 647** |
| --- |
| 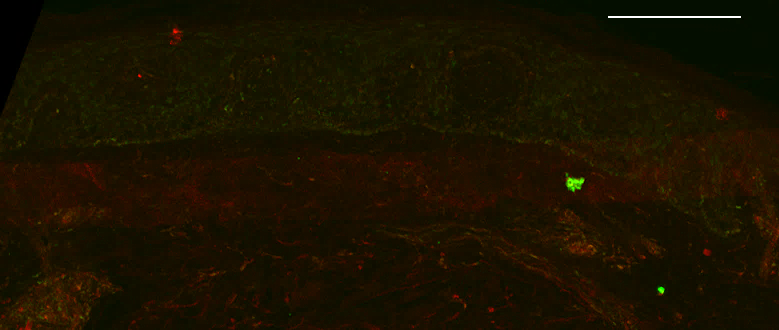 |
| 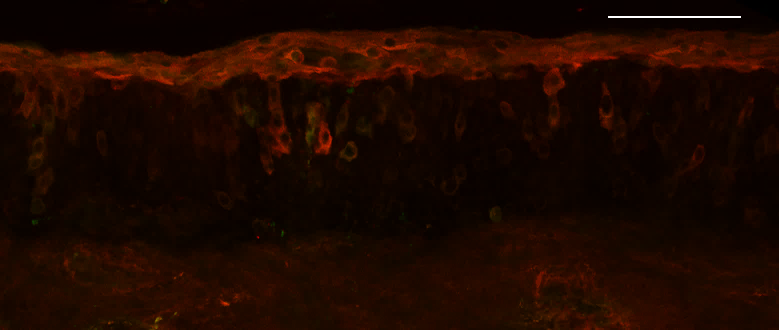  (c) CaMKK2 **DyLight® 594 AlexaFluor^TM^ 647** |
| **Supplementary Figure 1. Representative confocal images of negative control sections for P2X7R (a), P2X4R (b) and CaMKK2 (c) treated only with secondary antibodies DyLight® 594 and AlexaFluor^TM^ 647.**  Background staining of Dylight® 594 (red) and AlexaFluor^TM^ 647 (green) occurred in the dermis and blood vessels of all negative controls (a-c). Scale bar = 100μm |

| 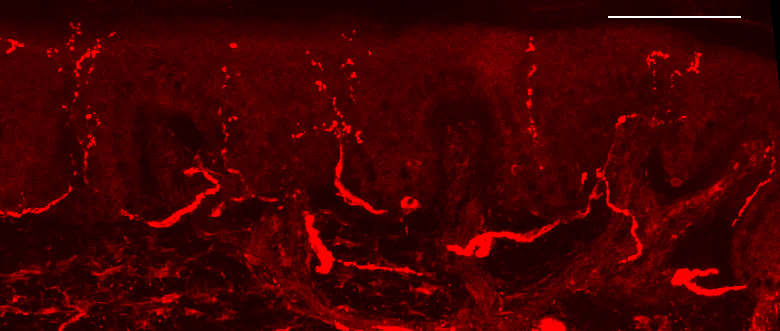  (a) **PGP9.5** |
| --- |
| 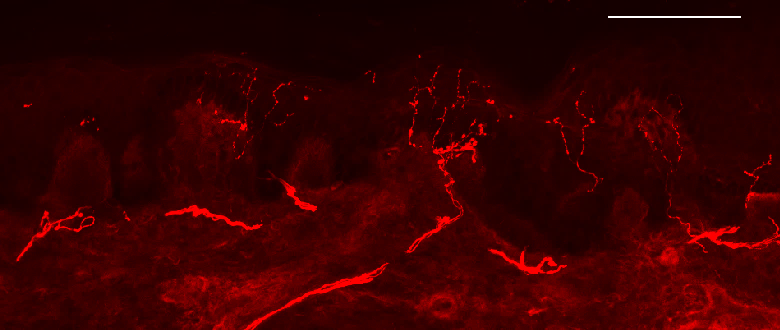  (c) **PGP9.5**  (b) **PGP9.5** |
| 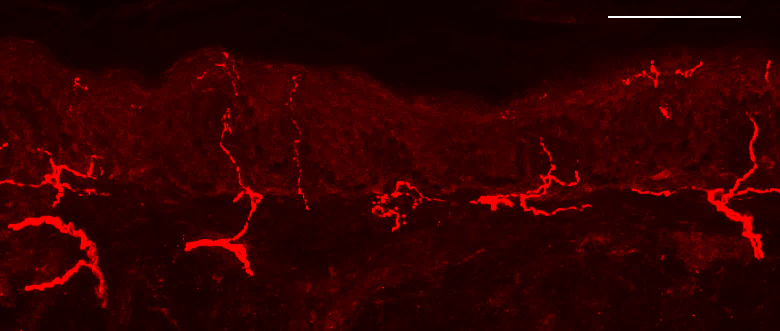 |
| **Supplementary Figure 2. Representative confocal images of PGP9.5+ intraepidermal nerve fibres from HC (a), HIV-SN- (b) and HIV-SN+ (c) donors.** Numerous PGP9.5+ fibres branch from dermal fibres innervating the epidermis in HC donors (a) with a median (range) intraepidermal nerve fibre density of 12.7 (7.4-17.3). The number of PGP9.5+ fibres were similar in HIV-SN– (5.2 [3.5-18.4]) and HIV-SN+ donors (3.8 [1.3-15.4]). A reduction in epidermal fibres length was common in HIV-SN+ donors (yellow arrows; c). Scale bar = 100μm |

**Supplementary Table 1. Expression of CaMKK2, P2X7R and P2X4R in all biopsies assessed**

| **#** | **HIV-SN^a^** | **IENFD (per mm2)** | **CaMKK2+ Cells  median (range)** | **P2X7R+ Cells  median (range)** | **P2X4R+ Brightness Score median (range)** |
| --- | --- | --- | --- | --- | --- |
| 1 | HC | 16.3 |  | 2 (0-2) | 3 (1-3) |
| 2 | HC | 16.0 | 0 (0) |  |  |
| 4 | HC | 12.7 | 2 (1-2) | 4 (1-4) | 1 (1-3) |
| 6 | HC | 10.2 |  |  | 2 (2-3) |
| 7 | HC | 7.4 | 2 (1-3) | 1 (0-3) |  |
| 9 | Neg | 11.3 | 8 (5-10) | 20 (19-24) |  |
| 10 | Neg | 18.4 |  |  | 3 (2-4) |
| 11 | Neg | 8.3 |  | 10 (4-12) | 2 (2-3) |
| 12 | Neg | 4.9 | 3 (1-6) |  |  |
| 13 | Neg | 3.9 | 5 (4-7) | 18 (18-26) |  |
| 16 | Neg | 12.4 |  |  | 2 (2-3) |
| 17 | Pos | 3.0 |  |  | 5 (4-5) |
| 18 | Pos | 15.4 |  | 4 (2-5) |  |
| 19 | Pos | 9.7 |  | 4 (2-8) |  |
| 20 | Pos | 2.8 | 13 (11-19) |  | 4 (4-5) |
| 23 | Pos | 10.0 | 8 (5-12) | 6 (3-7) |  |
| 24 | Pos | 1.3 | 17 (16-24) |  | 5 (4-5) |

1. HIV-SN diagnosis using BPNS

HC, healthy control; Pos, positive diagnosis; Neg, negative diagnosis; IENFD, intraepidermal nerve fibre density

A minimum of 3 images per section and 3 sections per donor were assessed by a single rater (JG) blinded to participant diagnoses. All CaMKK2+ and P2X7R+ cells were counted and the median (range) number of positive cells are reported. Most cells in the basal layer of the epidermis were P2X4R+ and therefore were scored using a brightness scale; 1, very weak; 2, weak; 3, moderate; 4, strong; 5, very strong. The median (range) brightness scores are reported.

**Supplementary Table 2. Clinical characteristics of all donors screened for IENFD**

| **#** | **HIV-SN^a^** | **Small fibre ^b^** | **Large fibre ^c^** | **Sex** | **Age (years)** | **Height (cm)** | **Nadir CD4 (cell/μL)** | **Last CD4 (cell/μL)** | **Time on ART (months)** | **IENFD (per mm^2^)** |
| --- | --- | --- | --- | --- | --- | --- | --- | --- | --- | --- |
| 1 | HC | - | - | Female | 26 | - | - | - | - | 16.3 |
| 2 | HC | - | - | Female | 41 | - | - | - | - | 16.0 |
| 3 | HC | - | - | Female | 30 | - | - | - | - | 17.3 |
| 4 | HC | - | - | Female | 33 | - | - | - | - | 12.7 |
| 5 | HC | - | - | Female | 33 | - | - | - | - | 9.7 |
| 6 | HC | - | - | Male | 37 | - | - | - | - | 10.2 |
| 7 | HC | - | - | Male | 23 | - | - | - | - | 7.4 |
| 8 | Neg | NA | Neg | Female | 40 | 150.5 | 251 | 448 | 76.8 | 5.1 |
| 9 | Neg | Neg | Neg | Female | 32 | 165 | 225 | 385 | 42.7 | 11.3 |
| 10 | Neg | Neg | Neg | Female | 36 | 153 | 234 | 321 | 84.4 | 18.4 |
| 11 | Neg | Pos | Neg | Female | 41 | 150 | 330 | 435 | 24.1 | 8.3 |
| 12 | Neg | Pos | Neg | Male | 31 | 174 | 214 | 386 | 58.6 | 4.9 |
| 13 | Neg | Neg | Neg | Male | 44 | 171 | 6 | 84 | 27.6 | 3.9 |
| 14 | Neg | Neg | Neg | Male | 38 | 179 | 179 | 626 | 103.7 | 5.2 |
| 15 | Neg | Pos | NA | Male | 35 | 165 | 28 | 693 | 140.7 | 3.5 |
| 16 | Neg | Pos | Neg | Male | 25 | 165 | 231 | 653 | 14.8 | 12.4 |
| 17 | Pos | Neg | Neg | Female | 37 | 155 | 43 | 526 | 92.2 | 3.0 |
| 18 | Pos | Neg | Neg | Female | 29 | 163 | 166 | 598 | 87.8 | 15.4 |
| 19 | Pos | Neg | Pos | Female | 32 | 167 | 117 | 729 | 47.4 | 9.7 |
| 20 | Pos | Pos | Pos | Female | 31 | 158 | 50 | 406 | 131.1 | 2.8 |
| 21 | Pos | Pos | Pos | Male | 47 | 167 | 17 | 284 | 80.9 | 4.6 |
| 22 | Pos | Neg | Neg | Male | 34 | 175 | 34 | 714 | 58.6 | 3.0 |
| 23 | Pos | Neg | Neg | Male | 41 | 171 | 143 | 444 | 110 | 10.0 |
| 24 | Pos | Neg | NA | Male | 45 | 167 | 32 | 300 | 24.4 | 1.3 |

1. HIV-SN diagnosis using BPNS
2. Small fibre neuropathy diagnosis using stimulated skin wrinkling tests (Safri et al).
3. Large fibre neuropathy diagnosis using nerve conduction tests (Safri et al).

HC, healthy control; NA, not available; Pos, positive test results; Neg, negative test result; ART, antiretroviral therapy; IENFD, intraepidermal nerve fibre density

**References**

Safri AY, Gaff J, Octaviana F, Setiawan DD, Imran D, Cherry CL, et al. Brief Report: Demographic and Genetic Associations With Markers of Small and Large Fiber Sensory Neuropathy in HIV Patients Treated Without Stavudine. J. Acquir. Immune Defic. Syndr. 2020;85:612-6.


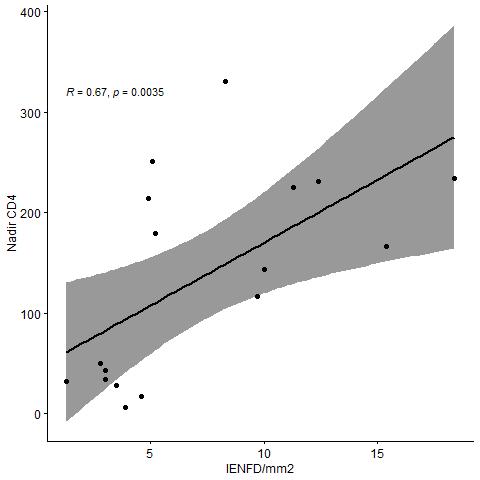


**Supplementary Figure 3. Nadir CD4 T-cell counts correlated directly with IENFD**

Spearman’s correlation coefficient

**Supplementary Table 3. IENFD in participants with and without neuropathy**

| **Diagnosis of Donors** | **IENFD (per mm^2^)** | | **P value** ^b^ |
| --- | --- | --- | --- |
|  | **Positive Donors ^a^** | **Negative Donors ^a^** |  |
| HIV-SN by BPNS | 3.8 (1.3-15.4) n=8 | 5.2 (3.5-18.4) n=9 | 0.19 |
| Large fibre neuropathy | 4.6 (2.8-9.7) n=3 | 8.0 (3.0-18.4) n=13 | 0.29 |
| Small fibre neuropathy | 4.8 (2.8-12.4) n=6 | 7.5 (1.3-18.4) n=10 | 0.62 |

^a^ Median (Range)

^b^ Mann-Whitney test
